# The Blackwall Tunnel from a Medical Point of View

**Published:** 1897-05-22

**Authors:** E. H. Snell

**Affiliations:** late London County Council Medical Officer at the Blackwall Tunnel


### Medical Progress and Hospital Clinics.

*The Editor will be glad to receive offers of co-operation and contributions from members of the profession. All letters should be addressed to* The Editor, at the Office, 28 & 29, Southampton Street, Strand, London, W.C.]

### THE BLACKWALL TUNNEL FROM A MEDICAL POINT OF VIEW.

By E. H. Snell, M.D., B.Sc.Lond., D.P.H., late London County Council Medical Officer at the Blackwall Tunnel.

The opening of the Blackwall Tunnel to-day by H.R.H. the Prince of Wales marks the completion, after six years of labour, of the greatest engineering feat of its kind that has ever been accomplished. It is not only of a larger diameter than any other tunnel made under water, but special difficulties depending on its proximity to the river bed, and the consequently imminent risk of failure, were anticipated and successfully overcome. The old Thames Tunnel at Wapping occupied Brunel for seventeen years before it was completed; nothing but his incomparable perseverance can receive the credit for that undertaking; with the resources which he had at his disposal the construction of the Blackwall Tunnel would have been even to Brunel an impossibility. The factor, which now assists us and which Brunel was without, is the use of compressed air for keeping out the water. Since 1839 compressed air has been used for this purpose in sinking shafts through water-bearing ground, and for forming the piles for bridges and piers, but it was not until 1879 that it was first employed in tunnel construction. In order to make use of this pneumatic method of tunnelling it is simply necessary to build a strong, air-tight partition somewhere in the course of the completed part of the tunnel, and then to force air by powerful engines into the portion in front of this partition; the air-pressure is then raised to a point when it is equal to the pressure of the water at the "face" where the men are working; if the soil being excavated is a porous one, like gravel, the air is continually escaping rapidly, and it has to be as rapidly replaced by the constant action of the engines. In this way the water is held back. The air-pressure necessary for this purpose will vary with the depth of the water to be combated. At Blackwall the highest pressure employed was 52 lbs. to the square inch (inclusive of the atmospheric pressure); there were thus three and a half times as much air in the chamber as normally. It is evident that to enter such a chamber through a door leading directly from the outside air would be a physical impossibility; the strongest iron door would be shattered before it would open inwards against such a pressure. The only method of providing means of ingress and egress is to build into the before-mentioned air-tight partition an "air-lock," with a door at each end opening towards the compressed air; then by an arrangement of taps the pressure of the lock can be raised or lowered gradually, and entry or exit respectively obtained, precisely as is the case with the water-lock of a canal.

The process of entry is often accompanied by severe pain in the ears until the knack of forcing air into the middle-ear by Valsalva's method is acquired. Should there be any unusual obstruction of the Eustachian tube this is often impossible, and then the warning pain is so severe that the attempt at entry has to be given up, or else the tympanic membrane will be ruptured. On this account a very slight nasal or pharyngeal catarrh is sufficient to prevent access to the air-chamber for a few days. In some cases the difficulty may be overcome by politzerisation; in others this is not effectual. When under these circumstances the attendance of a particular engineer suffering in this way is urgently required, it has been recommended that the membrane of the affected ear should be artificially punctured. The entry, although on this account often inconvenient and sometimes leading to inflammation of the middle ear and abscess, is not in itself dangerous, and the accidents thus caused are absolutely avoidable if the workmen are properly instructed.

The stay in the compressed air causes no inconvenience; one finds that it requires more muscular effort to put the air into vibration in speaking, the voice has a higher and more nasal tone, and it is difficult or impossible to whistle: except for these obvious peculiarities one would not know that anything is abnormal. But although the sojourn is, during its continuance, without apparent effect, the exit may be followed by serious illness or even by death. The occurrence of this illness has been noticed since compressed air was first employed as a working atmosphere, and many deaths have been due to its use. It is analogous to the illnesses to which divers are subject. The most common form in which it appears is that of pain in the extremities, known by the men as "bends"; this may supervene immediately on exit or within a few hours afterwards. The pain usually affects the legs, and principally the parts about the knees, though it may be present in the arms, especially the elbows and shoulders; it may be present in one or all of the limbs; its onset is usually sudden, becoming more and more severe for a short time afterwards. It may be only slight, causing little or no inconvenience, or it may be of a most excruciating character; sometimes it is a sharp and shooting pain, sometimes a dull, aching pain; it does not follow any anatomical distribution. There is generally some tenderness, and usually no swelling, though in a few rare cases tumefaction has existed, which has sometimes been followed by signs of ecchymosis. The pain, if slight, may pass off in a few hours, or it may last for weeks; it does not in itself appears to be dangerous, or to be followed by any sequelæ. A less common situation for the pain is the epigastrium, and this may be of great severity. Serious symptoms of collapse have been observed.

Sometimes the illness takes the form of paralysis of the legs, followed or not by the usual bladder and rectum troubles of a transverse myelitis; if no complications supervene the paralysis generally passes off by itself in from a few hours to a few months.

Or again, a form of acute Menière's disease may occur. Nine such cases were met with at the Blackwall Tunnel, and two of these have been most intractable to any form of treatment, although improvement has been gradual, and on general principles it would appear probable that the functions of equilibration of the damaged internal ear on the one side will ultimately be undertaken by that of the opposite side, as in other cases of acute auditory vertigo. Since, however, the affection as a result of compressed air has not been previously described and recognised, it appears at present best to await the result of experience. The resulting one-sided deafness in the more severe of these cases seems to be permanent, as also do the noises in the ear.

Hæmorrhage from the ears and nose has been often met with after leaving the compressed air; the cases, however, that have come under my own observation have, with one exception, been few and trivial. When death supervenes, it occurs either suddenly soon after exit, or else follows cystitis complicating paraplegia.

The discovery of the determining causes of the illness is of great importance, since it is only by obtaining a knowledge of them that effective preventive measures can be hoped for. So far as at present known, the three principal causes, with the variation of which the illness directly coincides are (1) the height of the pressure, (2) the length of stay in the compressed air, and (3) the lack of ventilation of the air-chamber. The first of these causes is apparent; the second was first pointed out by Mr. Eads, the chief engineer of the St. Louis Bridge in 1871; while the third was suggested and has been proved to be of paramount importance by observations made at the Blackwell Tunnel. There are two methods of gauging the ventilation of such an air space; either the air must be sampled in a large number of parts of the chamber and analysed several times daily, or else the amount of air supplied by the engines must be calculated; both methods are liable to some fallacies on account of the lessened diffusive power of compressed air. After a trial of both plans I decided that the latter was not only the most accurate but the most convenient, since, the revolutions of the engines being continuously recorded in the engine-room, the air supply at any moment, either past or present, could be immediately ascertained; the number of men in each shift being also recorded, the amount per head was known. The result has been a series of monthly tables, extending over a period of two years, which have shown conclusively that an excessive amount of ventilation coincided with a decrease in the amount of illness.

Other determining causes of the illness are: (4) A too rapid exit; and (5) personal idiosyncrasies. Thus stout men are more liable to the illness than thin men, older than younger men, and probably heavy drinkers than moderate drinkers or abstainers.

For over fifty years fresh theories concerning the pathology of the illness have been advanced. These may generally be grouped into three varieties: (1) Theories suggesting exhaustion, carbonic acid, and the like as the cause; (2) theories accounting for the symptoms by supposing congestion of different parts, mainly of the central nervous system; and (3) theories depending on an increased solution by the blood of the gases of the compressed air and the liberation of those gases on the pressure being removed.

Hitherto English and American authors have universally adhered to theories of the first or second variety. But I have elsewhere attempted to show that the truth will probably be found to lie in the increased solution of gases by the blood; and during the last twelve months Professor Schrötter, of Vienna, with whom I have been corresponding, has been carrying out a series of experiments on animals, which appear to place this matter beyond the possibility of doubt.

In adopting preventive measures the ætiological factors have as far as possible to be eliminated. At Blackwall the length of the stay for the workmen was eight hours, with an interval for a meal of three-quarters of an hour in the middle; with higher pressure shorter hours would probably be necessary. The ventilation must be very thorough, and should there not be a sufficiently free natural escape of air at the working "face," artificial outlets should be provided to prevent the ingoing air raising the pressure above what is actually required. A strict medical examination of new men is essential.

In the matter of remedies, the best method of cure is recompression, followed by a very slow exit. This was at Blackwall effected by means of a special medical lock or compressed air chamber placed in a convenient situation above ground, and used solely for this purpose. This remedy appears to be very effective when resorted to early; should it be delayed, little benefit is derived, and symptomatic treatment alone remains.

Owing to the numerous fatal accidents from compressed air previously recorded, it was feared that similar results might be encountered at Blackwall. Fortunately, although the illnesses were more numerous than might have been hoped for, they were in the main not of a serious character, and no fatal result followed. Subsequently to the completion of the compressed air work one case of death has been alleged to be due to this cause, but since the man had not worked under pressure for eleven months before his death, and the circumstances of the illness were such that its ascription to this cause would make it absolutely without parallel in the now numerous records of the illness, it has been impossible to accept it without grave doubt. The London County Council have, however, generously decided to overlook the medical difficulties of the case, and to help the widow as far as they have power.

To the Bridges Committee of the Council is largely due the credit for having suggested and supervised the methods of prevention of the illness attendant on this dangerous occupation with a thoroughness which has never before been attempted, and with a success which could scarcely be excelled.

